# Incidence, Pathogenesis, Risk Factors, and Treatment of Cystoid Macula Oedema Following Cataract Surgery: A Systematic Review

**DOI:** 10.3390/diagnostics15060667

**Published:** 2025-03-10

**Authors:** Lorenzo Ferro Desideri, Kirupakaran Arun, Enrico Bernardi, Nicola Sagurski, Rodrigo Anguita

**Affiliations:** 1 Department of Ophthalmology, Inselspital, Bern University Hospital, University of Bern, Freiburgstrasse 15, CH-3010 Bern, Switzerlandnicola.sagurski@insel.ch (N.S.); 2 Department for BioMedical Research, University of Bern, Murtenstrasse 24, CH-3008 Bern, Switzerland; 3 Bern Photographic Reading Center, Inselspital, Bern University Hospital, University of Bern, CH-3000 Bern, Switzerland; 4 Moorfields Eye Hospital NHS Foundation Trust, London EC1V 2PD, UK

**Keywords:** cystoid macular edema, postoperative edema, Irvine–Gass syndrome, pseudophakic

## Abstract

**Background/Objectives:** Cystoid macular edema (CMO) is a common complication that follows cataract surgery, presenting management challenges due to the lack of standardized treatment guidelines and the potential for spontaneous resolution. This study aimed to evaluate various treatment modalities for post-operative CMO, including topical non-steroidal anti-inflammatory drugs (NSAIDs), periocular steroids, and intravitreal injections. **Methods:** A systematic review of the literature was conducted to assess the efficacy of different treatment approaches for post-operative CMO. Studies evaluating topical NSAIDs, periocular steroids, intravitreal triamcinolone acetonide (TCA), dexamethasone implants (Ozurdex), and intravitreal bevacizumab were included. The main outcomes assessed included improvements in vision, resolution of CMO, recurrence rates, and safety profile. **Results:** Topical NSAIDs, particularly ketorolac and diclofenac, showed effectiveness in acute CMO, while their efficacy in chronic cases was variable. Periocular steroids, including retrobulbar TCA and sub-Tenon injections, demonstrated significant improvements in vision and the resolution of CMO, especially in cases resistant to topical therapy. Intravitreal TCA and dexamethasone implants exhibited variable effects on CMO resolution and recurrence rates, with some studies reporting sustained improvements over 12 months. The role of intravitreal bevacizumab as initial therapy remains unclear, although it may be considered in cases unresponsive to steroids. **Conclusions:** Topical NSAIDs, often combined with periocular steroids, serve as first-line therapy, with periocular steroids offering additional efficacy in resistant cases. Further research is needed to establish optimal treatment algorithms and improve outcomes for patients with post-operative CMO

## 1. Introduction

Cataract surgery is the most commonly performed outpatient surgery in developed countries, with an estimated 350,000 procedures performed in the United Kingdom and 3.7 million procedures in the USA and over 20 million worldwide in 2022 alone [1,2].

Modern-day cataract surgery produces improved visual acuity in over 95% of patients [3]; however, despite advances in cataract surgery, cystoid macula oedema (CMO) is still recognized as one of the most common causes of poor visual outcome following surgery [4]. In fact, CMO is characterized by the accumulation of a fluid within the macula and can lead to reduced visual acuity, distorted vision, and other visual disturbances [3,5]. Understanding its incidence, underlying pathogenesis, identifiable risk factors, and effective preventative strategies is crucial in optimizing patient outcomes and ensuring long-term visual health.

The aim of this review is to highlight the incidence, pathogenesis, risk factors, prophylaxis, and treatment of CMO.

## 2. Materials and Methods

**Figure 1:**
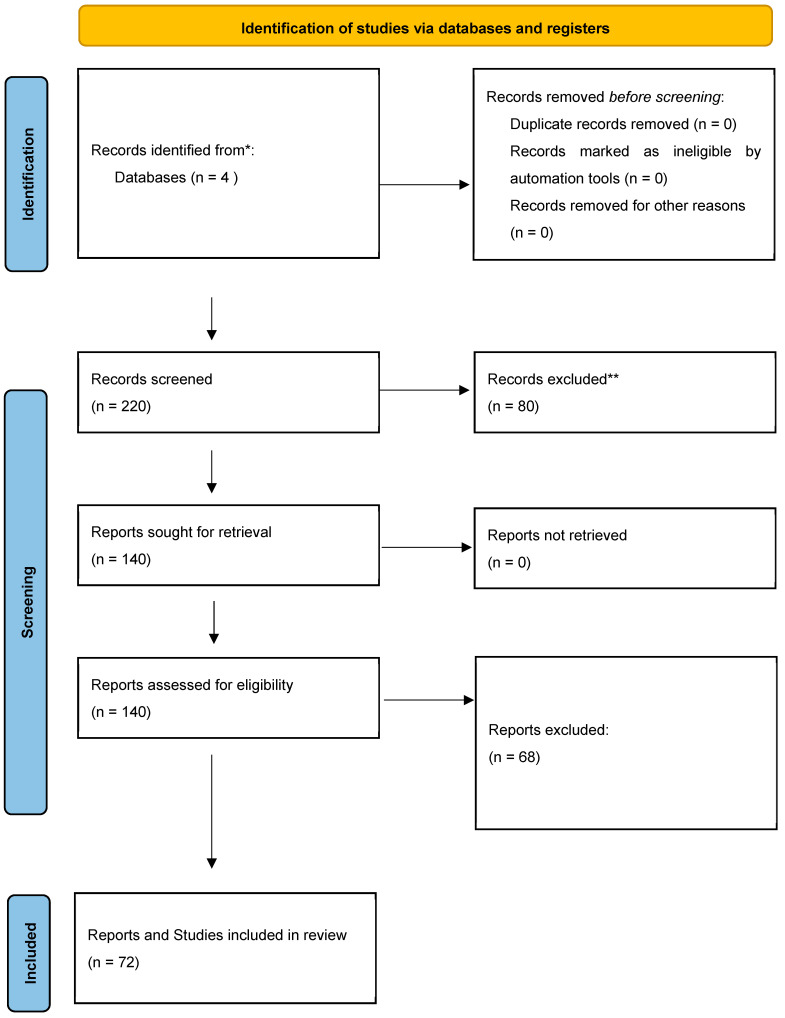
**PRISMA 2020 flow diagram for new systematic reviews that included searches of databases and registers only.** * Consider, if feasible to do so, reporting the number of records identified from each database or register searched (rather than the total number across all databases/registers). ** If automation tools were used, indicate how many records were excluded by a human and how many were excluded by the automation tools.

A comprehensive literature search was conducted to find all published studies on these topics from database inception until April 2024. The following databases were searched: Medline, PubMed, Web of Science Core Collection, and the Cochrane Library. Controlled vocabulary and keywords related to “macular edema” and “central macular edema” were used in combination with the terms “pseudophakic”, “cataract surgery”, and “postoperative”. No language or publication status restrictions were imposed. The authors thoroughly examined 220 retrieved records, selecting relevant articles for a full review based on their pertinence to the topic at hand. Additionally, the authors manually searched the reference lists of eligible articles to uncover further relevant studies. They also explored ongoing clinical trial registries, such as www.clinicaltrials.gov, to identify any research currently underway. This systematic review was performed in accordance with the PRISMA (Preferred Reporting Items for Systematic Reviews and Meta-Analyses) guidelines (Figure 1) and has not been registered in any publicly available registries.

## 3. Results

### 3.1. Definition and Incidence

CMO following cataract surgery was first reported in 1953 by Irvine et al. and was named Irvine–Gass syndrome [5]. It was defined as the presence of thickening of the macula, specifically after a cataract surgery. Since then, technological advances have led to different methods to diagnose and, thus, define CMO.

Post-operative CMO can be classified in three ways:

1. Angiographic CMO (based on angiographic findings from fundus fluorescein angiography),
2. Optical coherence tomography (OCT)-based CMO (from OCT findings of central subfield macular thickening),
3. Clinical CMO (from clinical findings of reduced visual acuity and findings on fundoscopy).

The incidence rates of CMO vary significantly according to the method of diagnosis and the type of cataract extraction technique (Table 1) [6,7,8,9,10,11,12,13,14,15,16,17,18]. The highest incidence is seen with angiographic CMO (16–60%), and the lowest incidence is with clinical CMO (0.1–20%). Furthermore, the incidence of post-operative CMO has been shown to be lower with less invasive and less traumatic cataract surgery approaches.

### 3.2. Clinical Presentation

The occurrence of CMO peaks at approximately 4–6 weeks following an uncomplicated cataract surgery [19]. Patients with symptomatic CMO can present with blurred vision, reduced contract sensitivity, central scotoma, and metamorphopsia [20].

CMO in which retinal thickening is greater than 300 μM can be visualized using a slit lamp or direct/indirect ophthalmoscopy as a loss of the foveal reflex. This is best seen using a green light to outline the cystic spaces. In some cases of clinical CMO, there may be associated faint vitreitis and optic nerve head swelling.

**Figure 2:**
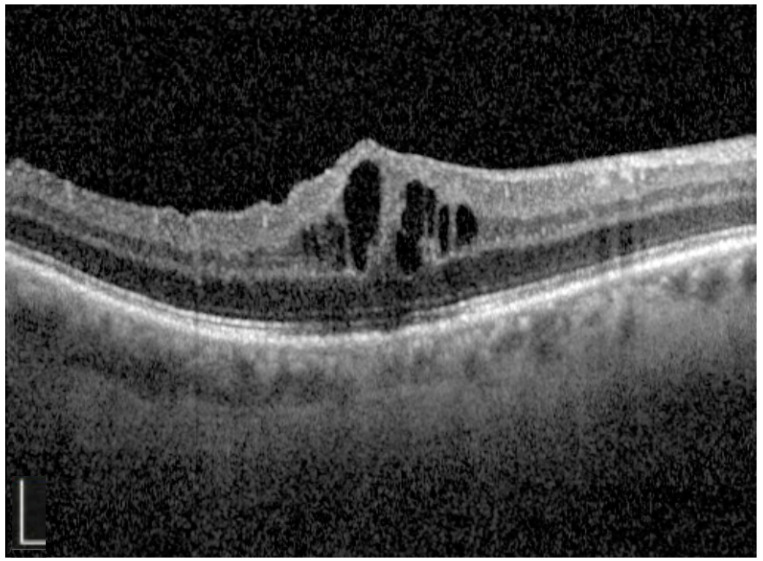
Optical coherence tomography image demonstrating localized foveal oedema and perifoveal cystic spaces that are seen in post-operative cystoid macula oedema. White bar 0.5 mm.

In many cases, there are no clinical signs of CMO, and this is best visualized with OCT or FFA. OCT imaging offers a minimally invasive, rapid, and risk-free way of both diagnosing and monitoring CMO [21]. The typical OCT findings of CMO are localized foveal oedema cystic spaces in the outer nuclear and outer plexiform layers (Figure 2) [22]. In severe cases of CMO, there is also detachment of the neurosensory retina with a subretinal fluid [23].

FFA plays an important role in differentiating post-operative CMO from other common causes of macular oedema such as diabetes and retinal vein occlusion. The features of angiographic CMO include leakage from the perifoveal capillaries in the early–mid phase that increase in size and intensity to form the classic “petaloid” appearance in the late phase [24]. Leakage from the capillaries of the optic disc is often seen in the late phase.

It should also be noted that angiographic CMO does not correlate well with reduced vision; however, OCT-based macular thickness measurements correlate much more closely with visual impairment [25,26].

More recent studies have found optical coherence tomography angiography (OCT-A) a useful tool in distinguishing post-operative CMO from DMO. Patients with post-operative CMO showed a more localized disruption of the parafoveal capillary arcade and cystoid spaces in the deep capillary plexus [27,28].

### 3.3. Pathogenesis

With CMO being first reported over 60 years ago, there have been various mechanisms implicated in its development. The earliest theory was that CMO was attributed to the incarceration of vitreous in the anterior segment and consequential vitreomacular traction, and as a result, initial treatment for CMO involved pars plana vitrectomy or neodyimium yttrium-aluminium-garnet (YAG) vitreolysis.

However, in 1966, there was a change in thinking towards an inflammatory process driving the development of post-operative CMO [29,30]. Gass performed fluorescein angiography on a series of patients that developed CMO and/or optic disc oedema following a cataract surgery, and this revealed a leakage of fluid from retinal and optic nerve head capillaries. The majority of these CMO patients were also found to have cells in the posterior vitreous.

This theory has been further emphasized by the observation that patients with more significant post-operative inflammation, longer operating times, and intra-operative complications have been shown to develop a high macular thickness that can last up to 6 months following surgery [31,32].

Since this time, with further understanding on the underlying pathological processes, post-operative inflammation has been universally agreed on as the major cause of the development of CMO following cataract surgery. It is believed that manipulation within the anterior chamber of the iris leads to the release of arachidonic acid from the uveal tissue. This, in turn, leads to the production of two inflammatory mediators: leukotrienes (via the lipoxygenase pathway) and prostaglandins (via the cyclooxygenase pathway) [33,34]. These inflammatory mediators diffuse posteriorly into the vitreous and towards the retina to cause a disruption of the blood–retinal barrier (through increased permeability of the perifoveal capillaries) [35]. Eosinophilic transudate accumulates in the outer plexiform and inner nuclear layers of the retina to create initial cystic spaces that become coalesced to form larger pockets of intra-retinal fluid [16].

It is thought that the high metabolic activity of the fovea and the lack of blood vessels within the avascular zone led to reduced fluid reabsorption near the macula to explain the leaking fluid accumulating at the fovea despite the more uniform distribution of leukotrienes and prostaglandins throughout the entire retina [16].

### 3.4. Risk Factors

There are numerous factors that have been shown to increase the risk of post-operative CMO, and these can be classified into systemic factors, ocular factors, and intra-operative factors. Table 2 provides a quantified summary of these risk factors.

It is well documented that the most significant systemic risk factor for the development of post-operative CMO is the presence of diabetes. In 2016, Chu et al. conducted the largest multicenter study to analyze this, with 81,984 consecutive phacoemulsification operations included [9]. They found that the eyes of diabetic patients who pre-operatively had no signs of diabetic eye disease (no macula oedema and no evidence of any Early Treatment Diabetic Retinopathy Study (ETDRS) grading of diabetic retinopathy (DRet)) were 1.8 times more likely to develop post-operative CMO than the eyes of patients without a diagnosis of diabetes.

There are numerous ocular risk factors associated with the development of CMO following a cataract surgery, with the most prevalent being the presence of pre-existing diabetic retinopathy. The risk of post-operative CMO significantly increased to 6.23 times more likely in the presence of any pre-operative DR, and there was a near-linear increase in risk dependent on the ETRDS-guided severity of DR.

Another study by Schimer et al. in 2007 analyzed 139,759 eyes in the United States that underwent cataract surgery and found similar results with a 1.78-times-higher chance of developing post-operative CMO in the eyes of diabetic patients compared to non-diabetic patients [36]. A more recent study by Patra et al. in 2017 analyzed 262 eyes in the United Kingdom that underwent phacoemulsification and found that there was a 3.06-times-higher incidence of post-operative CMO in the eyes of patients with a history of diabetes compared to eyes without a history of diabetes [37]. These findings are all consistent with the theory that CMO is driven by an inflammatory process that impairs the blood–retinal barrier, as diabetic patients are known to have more advanced retinal vascular changes from DR.

Ethnicity was not found to be an independent risk factor in the development of post-operative CMO. However, it is well established that there is a higher prevalence of DR and sight threatening DR in minority ethnic groups (South Asian, African, and Afro-Caribbean), and this correlates well with the higher rates of CMO seen in minority ethnic groups in clinical practice [37,43].

Given the inflammatory processes that underpin the development of CMO following a cataract surgery, it is unsurprising that uveitis is a known risk factor for the development of post-operative CMO. Chu et al. found that eyes with a previous history of uveitis were 2.88 times more likely to develop CMO than eyes with no previous uveitis. A different study found similar results in that patients with previous uveitis were 3.00 times more likely to develop CMO than patients with no previous uveitis [38]. Eyes with active uveitis in the 3-month period prior to surgery had a significantly higher incidence of CMO at 1 month (38%) compared to eyes with previous episodes of uveitis but no active inflammation in the 3 months prior to surgery (6%) and compared to eyes with no previous uveitis (4%).

Previous retinal vein occlusion is another well-documented risk factor for CMO. Chu et al. demonstrated that patients with a previous retinal vein occlusion were 4.47 times more likely to develop CMO following a cataract surgery. Very similar results were produced by Henderson et al., who found that patients with a retinal vein occlusion were 4.64 times more likely to develop CMO [9,39]. In both studies, there was confirmed absence of macula oedema prior to cataract surgery. Other factors associated with an increased risk of developing post-operative CMO include previous treatment for macular oedema and a high number of previous intravitreal injections [44].

The presence of a pre-existing epiretinal membrane (ERM) has been shown to also have a significant effect on increasing the risk of post-operative CMO. Chu et al. found that patients with a pre-existing ERM were 5.6 times more likely to develop post-operative CMO [9]. Schaub et al. also found a relative risk: the development of post-operative CMO was increased in eyes with previously diagnosed ERM by 2.67 [40].

Numerous studies have identified previous vitrectomy for retinal detachment as an important risk factor for the development of CMO following a cataract surgery [9,40,45,46]. However, one of the ocular factors where the evidence is less clear is the use of topical glaucoma medications. The largest study to date (which included 3394 eyes with prostaglandin use prior to a cataract surgery) found no increase in the risk of the development of post-operative CMO [9]. Law et al. also found similar results with no association between pre-operative glaucoma drops and the presence of post-operative clinical CMO [41].

Other studies (both retrospective and prospective) have found pre-operative prostaglandin use to be associated with an increased risk of post-operative CMO [39,47,48]. However, all of these studies used angiographic CMO as their primary outcome measure and did not assess for clinical CMO and, thus, may not be relevant to clinical practice.

Looking at intra-operative factors, the use of pupil expansion devices and intra-operative posterior capsule rupture have both been demonstrated to increase the risk of development of post-operative CMO [9,39,41,42]. This is in keeping with the theory that increased manipulation in the anterior chamber is likely to precipitate the release of more pro-inflammatory mediators that then diffuse towards the retina and cause disruption to the blood–retinal barrier.

### 3.5. Prophylaxis

With a greater understanding of the processes that underpin the development of post-operative CMO, there are various strategies to help prevent CMO and these are all aimed at controlled postoperative inflammation and targeting the ocular and intra-operative risk factors. The intraocular risk factors are addressed by performing the least invasive surgery, with no complications and a shorter operating time. There is no clear universal practice employed to target the ocular risk factors, but it usually involves the use of anti-inflammatory pharmacological agents. Table 3 summarizes recent relevant publications regarding the prevention of CMO in cataract surgery.

#### 3.5.1. Topical NSAIDs Prior to Surgery

Some authors have found benefits in administering topical anti-inflammatories prior to surgery. The two largest studies that analyzed this both found that pre-operative topical NSAIDs (non-steroidal anti-inflammatory drugs) decreased the incidence of post-op CMO compared to placebo or no pre-operative topical treatment. Donnenfeld et al. assessed randomized eyes into Groups 1 of 4 based on different preoperative dosing regimens of ketorolac 0.4%. Group 1 received ketorolac for 3 days prior to surgery, Group 2 for 1 day, and Group 3 for 1 h prior to surgery, while Group 4 received placebo [49]. Groups 1–3 received prednisolone and ketorolac post-operatively, and Group 4 received prednisolone and placebo. They found that visual acuity was better for Groups 1 and 2 at 2 weeks, but this was not sustained at 3 months.

A larger study by Yavas et al. analyzed the effectiveness of 3 days of a different pre-operative topical NSAID (indomethacin 0.1% 1 drop administered 4 times daily) and found a significant reduction (*p* = 0.001) in angiographic CMO at 3 months compared to eyes that did not receive any pre-operative treatment [50]. Both groups received the same post-operative drops.

#### 3.5.2. Topical NSAIDs After Surgery

Many institutions utilize post-operative NSAIDs in addition to topical steroids in order to reduce the risk of post-operative CMO. However, the literature is not clear if there is a clear benefit.

Two systematic reviews compared post-operative NSAIDs against topical post-operative steroids and found that the NSAID group had less angiographic and OCT-based CMO and faster initial visual recovery [51,52]. However, both studies also found no significant difference in visual acuity between the 2 groups at 3 months.

Two other systematic reviews compared the effect of topical NSAID alone or in addition to topical corticosteroids against topical corticosteroids alone and found very low evidence to suggest the superiority of post-operative NSAIDs with or without adjunctive corticosteroids in reducing the incidence of post-operative CMO [53,54]. By contrast, two recent randomized control trials found that adding a prophylactic post-operative NSAID to steroid resulted in lower incidences of clinical CMO at 3 months [55,56].

When comparing specific post-operative topical NSAIDs against each other, there are varying results. In an RCT by Almeida et al., there was no difference in post-operative OCT-based CMO at 1 month between nepafenac 0.1% and ketorolac 0.5% [57]. Lee et al. also found no difference at 1 month in OCT-based CMO between diclofenac 0.1% and ketorolac 0.45%, but there was a significantly lower incidence of OCT-based CMO in the ketorolac group at 2 months [58]. Wang et al. found that bromfenac 0.1% produced lower rates of OCT-based CMO at 3 months than fluoromethalone 0.1%, but there was no difference in clinical CMO [59].

#### 3.5.3. Intra-Operative Treatment in Diabetic Eyes

A recent randomized control trial was conducted to clarify the optimal treatment in diabetic eyes [60]. Eyes were randomized to receive either no additional treatment, 40 mg triamcinolone subconjunctival injection, 1.25 mg intravitreal bevacizumab injection, or a combination of both injections. All patients received the same pre- and post-operative topical treatments (steroid and NSAID). They found that OCT-based macular thickness and volume at 6 and 12 weeks post-operatively differed in the triamcinolone group when comparing eyes that received no treatment and eyes that received intravitreal bevacizumab, but no difference in visual acuity was observed in any of the groups.

#### 3.5.4. Topical NSAIDs After Surgery in Diabetic Eyes

Whilst the consensus is that there is minimal benefit in adding post-operative topical NSAIDs to steroids for low-risk cases, there is more clear evidence to suggest a benefit in diabetic eyes. Various randomized control trials have found a reduction in both OCT-based and clinical CMO in diabetic eyes when using adjuvant nepafenac, diclofenac 0.1%, and ketorolac 0.5% [39,61].

#### 3.5.5. Topical NSAIDs Before and After Surgery in Diabetic Eyes

The use of both pre- and post-operative topical NSAIDs in diabetic eyes was assessed by Singh et al. [62]. They randomized eyes into two groups: nepafenac or placebo three times daily beginning 1 day prior to surgery and through day 90 following surgery. They found significantly lower incidences of both clinical and OCT-based CMO in the nepafenac group, suggesting the clinical benefits of such a regime in higher-risk diabetic eyes.

### 3.6. Treatments and Outcomes

Due to the sheer number of cataract surgeries performed, clinical CMO remains a common issue that is faced by all ophthalmologists. Despite this, there are no universally accepted guidelines regarding treatment strategies. One of the major confounding factors in evaluating the success of any treatment is the fact that clinical CMO often resolves spontaneously, and as such, the true effect of any treatment can be difficult to quantify [63]. Table 4 contains evidence of findings regarding treatment for post-phacoemulsification CMO from the literature.

#### 3.6.1. Topical Treatment

Sivaprasad et al. performed a systematic review of the efficacy of different topical NSAIDs for the treatment of post-operative acute and chronic CMO [64]. Regarding acute CMO (defined as oedema of less than four months), they found that a combined treatment with ketorolac and corticosteroid and monotherapy with either ketorolac or diclofenac were all effective. Their review found mixed results regarding topical NSAIDs for chronic CMO. Two trials demonstrated that topical 0.5% ketorolac had a positive effect on chronic CMO, with three months of treatment showing better therapeutic response. One trial found no benefits with oral indomethacin, and one trial found some benefits (but not statistically significant) with topical fenoprofen. They also found that an improvement in angiographic CMO did not correlate with improvements in vision.

#### 3.6.2. Periocular Steroids

For cases of CMO that do not respond to topical therapy, periocular steroids can be considered. A retrospective cohort study analyzed eyes with post-op CMO that failed to resolve with topical treatment and were treated with either a single retrobulbar triamcinolone (TCA) injection or three bi-weekly Sub-Tenon TCA injections. Both treatment approaches resulted in significant improvement in vision and the resolution of clinical CMO with no concerns with intraocular pressure rise [65].

One prospective study compared topical nepafenac with a single sub-Tenon TCA injection and found improvements in both clinical and OCT-based CMO but a larger effect with topical nepafenac [67], which strongly points towards trialing topical nepafenac in patients with refractory CMO as a first-line therapy. Orbital floor 40 mg TCA has been showed to significantly reduce OCT-based CMO as well as produce improvements in visual acuity, although no large-sample-size studies have been conducted [66].

#### 3.6.3. Intravitreal Steroid Injection

Intravitreal TCA has shown variable effects regarding refractory CMO. One study found improvements in both OCT-based and clinical CMO that was maintained at 12 months. However, another study found that repeated intravitreal TCA showed recurrence of CMO at 4 months [68,69]. Neither study showed any rise in intraocular pressure.

#### 3.6.4. Intravitreal Dexamethasone Implant (Ozurdex)

Two studies found positive treatment effects with dexamethasone implants that lasted for approximately 3 months. Bellocq et al. found Ozurdex implants to be effective in treating eyes with CMO unresponsive to topical therapy, with improvements in clinical CMO that were maintained at 12 months [70]. A total of 37% of the eyes only required a single Ozurdex implant. Very similar results were obtained by Meyer et al., who found that 61% of eyes required only a single Ozurdex implant [71].

#### 3.6.5. Intravitreal Bevacizumab

The role of intravitreal anti-VEGF (vascular endothelial growth factor) injections in the treatment of CMO that does not respond to first-line topical NSAID therapy is not clear. Falavarjani et al. reviewed 11 studies that used intravitreal bevacizuamab for the treatment of post cataract surgery CMO and did not find high-quality evidence to recommend it as an initial treatment but said it could be considered if unresponsive to intravitreal steroids [72].

## 4. Discussion

Acute and chronic CMO remains a common and potentially visually debilitating post-operative complication seen in cataract surgery. CMO can be defined based on clinical signs and symptoms: primarily based on OCT and secondarily based on FFA. In modern-day clinical practice, OCT provides the safest, quickest, and most reliable tool to both diagnose and monitor CMO.

The complex pathogenesis of CMO still needs to be fully understood, but the major driving force appears to arise from inflammation from intra-operative manipulation within the anterior chamber.

The risk factors for the development of post-operative CMO have been clearly identified from the literature, and the systemic and ocular factors such as diabetes, diabetic retinopathy, and uveitis should be identified pre-operatively to allow for appropriate consent and planning.

Prophylaxis with topical NSAIDs appears to show benefits in high-risk patients such as patients with a history of diabetes and eyes with diabetic retinopathy, pre-existing epiretinal membranes, or previous intraocular inflammation, although there is no consensus on when this should be initiated or the recommended treatment regime.

Despite the enormous amount of cataract surgeries performed, there is no standardized guidelines on the treatment of post-operative CMO. Universal first-line treatment appears to be topical NSAIDs, either as monotherapy or in combination with topical corticosteroids; however, while NSAIDs have shown efficacy, particularly in acute CMO, their effectiveness in chronic cases is less clear. Periocular steroids, including retrobulbar and sub-tenon injections, have demonstrated significant improvements in vision and the resolution of CMO, especially in cases refractory to topical therapy. However, the choice between topical NSAIDs and periocular steroids as first-line therapy requires careful consideration of individual patient factors and response to initial treatment. If the CMO does not respond to the first-line therapy, various other treatment options have been explored and appear to show benefits, such as periocular TCA, intravitreal steroid, and intravitreal anti-VEGF agents.

The role of intravitreal anti-VEGF injections, such as bevacizumab, as initial therapy remains uncertain but may be considered in cases unresponsive to steroids.

## 5. Conclusions

Due to the varying definitions of CMO, there is a lack of consistent conclusions regarding prophylaxis and treatment in systematic reviews. As such, high-quality, prospective randomized control trials are still required to allow for the development of evidence-based standardized prophylaxis and treatment protocols. So far, universal first-line treatment appears to be topical NSAIDs, either as monotherapy or in combination with topical corticosteroids.

## Figures and Tables

**Table 1:** Incidence of CMO at 4–16 weeks following uncomplicated cataract surgery.

Cataract Extraction Technique	Incidence of Clinical CMO (%)	Incidence of OCT-Based CMO (%)	Incidence of Angiographic CMO (%)
Intracapsular cataract extraction	8 [6]	Not available	36–60 [13]
Extracapsular cataract extraction	0.8–20 [7]	Not available	16–32.2 [14,15,16]
Phacoemulsification	0.1–2.35 [8,9]	3–41 [11,12]	20–54.7 [17,18]
Femtosecond-assisted cataract surgery	1.18 [10]	1.18 [10]	Not available

Abbreviations: CMO, cystoid macula oedema; OCT (optical coherence tomography).

**Table 2:** Risk factors for developing CMO following cataract surgery.

Authors	Risk Factor	Level of Evidence	Study Type (No. of Eyes)	Follow-Up (Months)	Incidence of CMO (%)	Relative Risk (95% Confidence Interval)
Systemic Risk Factors						
Diabetes Mellitus (DM)						
Chu et al., 2016 [9]	DM without DR	III-2	Retrospective (81,984)	3	2.15	1.80 (1.38–2.36)
Ocular Risk Factors						
Diabetes Mellitus (DM)						
Chu et al., 2016 [9]	DM with any DR	III-2	Retrospective (81,984)	3	7.27	6.23 (5.12–7.58)
	DM with stable PDR and previous PRP				10.63	9.11 (6.07–13.68)
	DM with PDR				12.07	10.34 (5.13–20.85)
	DM with severe NPDR				7.69	6.59 (2.21–19.63)
	DM with moderate NPDR				9.95	8.53 (5.62–12.93)
	DM with mild NPDR				9.43	8.08 (6.03–10.85)
Schimer et al., 2007 [36]	DM	III-2	Retrospective (139,759)	12	3.05	1.77 (1.62–1.92)
Patra et al., 2017 [37]	DM	III-2	Retrospective (262 eyes)	12	10.7	3.06
Uveitis						
Chu et al., 2016 [9]	Uveitis	III-2	Retrospective (81,984)	3	3.36	2.88 (1.50–5.51)
Belair et al., 2009 [38]	Previous non-infectious uveitis	III-2	Prospective (93)	3	12	3.11 (0.64–15.20)
	Previous uveitis, but no active uveitis within 3 months of surgery				6	1.55
	Previous uveitis and active uveitis within 3 months of surgery				38	9.87
Retinal Vein Occlusion (RVO)						
Chu et al., 2016 [9]	Previous RVO	III-2	Retrospective (81,984)	3	5.22	4.47 (2.56–7.82)
Henderson et al., 2007 [39]	Previous RVO	III-2	Retrospective (1659)	12	10.3	4.64
Epiretinal membrane (ERM)						
Chu et al., 2016 [9]	Pre-existing ERM	III-2	Retrospective (81,984)	3	6.53	5.60 (3.45–9.07)
Henderson et al., 2007 [39]	History of ERM	III-2	Retrospective (1659)	12	7.14	3.21 (1.03–10.00)
Schaub et al., 2018 [40]	Pre-existing ERM	III-2	Retrospective (357)	11	15.69	2.67 (1.31–5.42)
Retinal Detachment (RD)						
Chu et al., 2016 [9]	Previous RD repair	III-2	Retrospective (81,984)	3	4.58	3.93 (2.60–5.92)
Schaub et al., 2018 [40]	Vitrectomy for RD	III-2	Retrospective (357)	11	28.13	2.86 (1.50–5.44)
Glaucoma medications						
Chu et al., 2016 [9]	Pre-op prostaglandin use	III-2	Retrospective (81,984)	3	1.30	1.11 (0.82–1.51)
Henderson et al., 2007 [39]	Historyof ERM	III-2	Retrospective (1659)	12	3.4	1.53
Intra-Operative Risk Factors						
Posterior Capsular Rupture (PCR)						
Chu et al., 2016 [9]	PCR +/− vitreous loss	III-2	Retrospective (81,984)	3	3.05	2.61 (1.57–4.34)
Law et al., 2010 [41]	PCR + vitrectomy	III-2	Retrospective (1253)	3	15	2.94 (1.35–6.38)
Henderson et al., 2000 [39]	PCR +/− vitreous loss	III-2	Retrospective (1659)	12	12.8	5.77
Use of pupil expansion device						
Taipale et al., 2019 [42]	Malyugin Ring used	III-2	Prospective (536)	3	15.0	5.41 (1.35–21.71)

Abbreviations: CMO (cystoid macula oedema), DR (diabetic retinopathy), PDR (proliferative diabetic retinopathy), NPDR (non-proliferative diabetic retinopathy), PRP (pan retinal photocoagulation).

**Table 3:** Prevention of post-operative cystoid macula oedema (CMO) following cataract surgery.

Authors	Level of Evidence	Study Type (No. of Eyes)	Intervention	Conclusions
Pre-Operative NSAID in Non-Diabetics				
Donnenfeld et al., 2006 [49]	II	RCT (100)	Ketorolac 0.4% prior to surgery for 1 h 1 day or 3 days	Ketorolac given for 1 or 3 days pre-operatively improves post-operative visual acuity at 2 weeks Not sustained at 3 months
Yavas et al., 2007 [50]	II	RCT (179 eyes)	Indomethacin 0.1% 1 drop 4 times daily for 3 days	Significant reduction in angiographic CMO at 3 months
Post-Operative NSAID in Non-Diabetics				
Kim et al., 2017 [51]	I	Systematic review	NSAID vs. steroid	Post-operative NSAID is effective in reducing angiographic and OCT-based CMO compared to topical corticosteroid NSAID alone produces faster visual recovery compared to steroid alone No difference in clinical CMO or visual acuity at 3 months
Kessel et al., 2014 [52]	I	Systematic review	NSAID vs. steroid	Post-operative NSAID is effective in reducing angiographic and OCT-based CMO compared to topical corticosteroid No difference in clinical CMO at 3 months
Lim et al., 2016 [53]	I	Systematic review	NSAID vs. steroid alone vs. NSAID + steroid	Low evidence of CMO reduction with NSAID alone
Juthani et al., 2018 [54]	I	Systematic review	NSAID vs. steroid alone vs. NSAID + steroid	Low evidence of any benefits of adding NSAID to post-operative steroid in reducing CMO
Wielders et al., 2018 [55]	II	RCT (914 eyes)	NSAID vs. steroid alone vs. NSAID + steroid	Combination post-operative regime reduces the incidence of clinical CMO compared to monotherapy
Shorstein et al., 2015 [56]	III	Retrospective cohort (16,070 eyes)	NSAID vs. NSAID + steroid	Adding post-operative NSAID reduced the incidence of clinical CMO
Comparing Post-Operative NSAID in Non-Diabetics				
Almeida et al., 2012 [57]	II	RCT (162 eyes)	Ketorolac 0.5% vs. nepafenac 0.1%	No difference in OCT-based or clinical CMO
Lee et al., 2015 [58]	III	Retrospective cohort (76 eyes)	Ketorolac 0.45% vs. diclofenac 0.1%	No difference in OCT-based or clinical CMO at 1 month Significantly less OCT-based CMO at 2 months in ketorolac group
Wang et al., 2013 [59]	II	RCT (167 eyes)	Bromfenac 0.1% vs. fluoromethalone 0.1%	Bromfenac resulted in less OCT-based CMO but no difference in clinical CMO at 3 months
Intra-Operative Treatment in Diabetics				
Wielders et al., 2018 [60]	III	RCT (213 eyes)	Subconjunctival 40 mg triamcinolone vs. intravitreal 1.25 mg bevacizumab vs. no treatment	Subconjunctival triamcinolone resulted in significantly lower incidence of OCT-based CMO at 6 and 12 weeks No difference in visual acuity between 3 groups
Post-Operative NSAID in diabetics				
Henderson et al., 2007 [39]	III	Retrospective cohort (1659)	Ketorolac 0.5% or diclofenac 0.1%	Both resulted in significant reduction in incidence of OCT-based and angiographic CMO to resemble incidence in low-risk eyes
McCafferty et al., 2017 [61]	II	RCT (1000 eyes)	Nepafenac 0.3% vs. placebo	Nepafenac resulted in significant reduction in incidence of clinical, OCT-based, and angiographic CMO
Pre and Post-Operative NSAID in Diabetics				
Singh et al., 2012 [62]	II	RCT (263 eyes)	Nepafenac vs. placebo from day 1 prior to surgery to day 90 post surgery	Nepafenac resulted in significant reduction in incidence of clinical and OCT-based CMO

Abbreviations: CMO (cystoid macula oedema); NSAID (non-steroidal anti-inflammatory drug); OCT (optical coherence tomography); RCT (randomized control trial).

**Table 4:** Treatment of post-operative cystoid macula oedema (CMO) following cataract surgery.

Authors	Level of Evidence	Study Type (No. of Eyes)	Intervention	Conclusions
Topical Therapy				
Sivaprasad et al., 2012 [64]	I	Systematic review	Topical NSAID, oral NSAID	Acute CMO Tends to resolve spontaneously Topical NSAIDs alone, topical steroid alone, or NSAID + steroid combination all effective Chronic CMO 3 months of topical 0.5% ketorolac had significant reduction in OCT, angiographic, and clinical CMO No effect with oral indomethacin Some improvements with topical fenoprofen but not significant
Periocular Steroids				
Thach et al., 1997 [65]	III	Retrospective cohort (49 eyes)	Retrobulbar TCA vs. Subtenons TCA	Both produced significant reduction in OCT- based and clinical CMO No difference between treatments
Suleman et al., 2008 [66]	IV	Case series (6 eyes)	Orbital floor TCA	Reduced retinal thickness in all eyes Improved visual acuity in 83% of eyes
Periocular Steroid vs. Topical NSAID				
Yuksel et al., 2017 [67]	III	Prospective randomized (48 eyes)	Nepafenac drops vs. single sub-Tenons triamcinolone	Both produced significant reduction in OCT- based and clinical CMO Larger effect with topical nepafenac
Intravitreal Steroid Injection				
Koutsandrea et al., 2007 [68]	IV	Retrospective case series (14 eyes)	Intravitreal TCA	Reductions in OCT-based and clinical CMO that was maintained at 12 months
Benhamou et al., 2003 [69]	IV	Prospective case series (3 eyes)	Intravitreal 8 mg TCA	Initial reductions in OCT-based and clinical CMO at 1 month, but back to baseline by 2–4 months
Intravitreal Dexamethasone Implant				
Bellocq et al., 2017 [70]	IV	Retrospective (58 eyes)	Ozurdex implant	Improvements in OCT-based and clinical CMO that were maintained at 12 month.
Mayer et al. [71]	III	Prospective case series (23 eyes)	Ozurdex implant	Improvements in OCT-based and clinical CMO that were maintained at 12 month.
Intravitreal Bevacizumab				
Falavarjani et al., 2012 [72]	I	Systematic review	Intravitreal bevacizumab	Inconclusive but can consider if no response to intravitreal steroids

Abbreviations: CMO (cystoid macula oedema); NSAID (non-steroidal anti-inflammatory drug); OCT (optical coherence tomography); TCA (triamcinolone).

## Data Availability

All the data are conserved by Inselspital University Hospital, Bern (Switzerland), in a private server.
